# Climate Change and Human Activities, the Significant Dynamic Drivers of Himalayan Goral Distribution (*Naemorhedus goral*)

**DOI:** 10.3390/biology12040610

**Published:** 2023-04-18

**Authors:** Shiekh Marifatul Haq, Muhammad Waheed, Riyaz Ahmad, Rainer W. Bussmann, Fahim Arshad, Arshad Mahmood Khan, Ryan Casini, Abed Alataway, Ahmed Z. Dewidar, Hosam O. Elansary

**Affiliations:** 1 Department of Ethnobotany, Institute of Botany, Ilia State University, 0162 Tbilisi, Georgia; rainer.bussmann@iliauni.edu.ge; 2 Department of Botany, University of Okara, Okara 56300, Pakistan; f19-phd-bot-5013@uo.edu.pk (M.W.); fahim.arshad@uo.edu.pk (F.A.); 3 National Center for Wildlife, Riyadh 11575, Saudi Arabia; riyaz.cf@gmail.com; 4 Department of Botany, Institute of Life Sciences, State Museum of Natural History, 76133 Karlsruhe, Germany; 5 Department of Botany, Government Hashmat Ali Islamia Associate College Rawalpindi, Rawalpindi 46300, Pakistan; arshadbotanist@gmail.com; 6 Department of Botany, Pir Mehr Ali Shah Arid Agriculture University Rawalpindi, Rawalpindi 46300, Pakistan; 7 School of Public Health, University of California, Berkeley, 2121 Berkeley Way, Berkeley, CA 94704, USA; ryan.casini@berkeley.edu; 8 Prince Sultan Bin Abdulaziz International Prize for Water Chair, Prince Sultan Institute for Environmental, Water and Desert Research, King Saud University, Riyadh 11451, Saudi Arabia; aalataway@ksu.edu.sa (A.A.);; 9 Department of Agricultural Engineering, College of Food and Agriculture Sciences, King Saud University, Riyadh 11451, Saudi Arabia; 10 Plant Production Department, College of Food & Agriculture Sciences, King Saud University, Riyadh 11451, Saudi Arabia

**Keywords:** Himalayan goral (*Naemorhedus goral*), conservation, climate change, species distribution modeling

## Abstract

**Simple Summary:**

The objective of our study was to gain a deep understanding about the role of predicted climate change on the range restricted and high-mountain dwelling animal species like the Himalayan goral (HG). To achieve this, we conducted species distribution modeling of the target species under present and predicted future climate change scenarios. Our focus was on identifying the potential suitable microhabitats of the target species using a predictive modeling and maximum entropy algorithm under current climate, and any possible range variation and shift under future climate change scenarios. Annual precipitation and elevation are important factors that affect goral distribution; the elevation between 2000 and 3000 m is thought to be the most suitable for the goral’s habitat. Additionally, a general slight range shift toward northern latitudes and along the higher elevations is observed. Our analysis shows that the suitability of the Himalayan goral’s habitat can change dramatically depending on the forecast. This work might fill the existing knowledge gap about the target species distribution in the study area and predict suitable habitats necessary for species conservation. This study might also be helpful in future planning, management, and sustainable use of available resources, and to mitigate the possible negative effects of climate change in the Himalayan region.

**Abstract:**

The distribution of large ungulates is more often negatively impacted by the changing climate, especially global warming and species with limited distributional zones. While developing conservation action plans for the threatened species such as the Himalayan goral (*Naemorhedus goral* Hardwicke 1825; a mountain goat that mostly inhabits rocky cliffs), it is imperative to comprehend how future distributions might vary based on predicted climate change. In this work, MaxEnt modeling was employed to assess the habitat suitability of the target species under varying climate scenarios. Such studies have provided highly useful information but to date no such research work has been conducted that considers this endemic animal species of the Himalayas. A total of 81 species presence points, 19 bioclimatic and 3 topographic variables were employed in the species distribution modeling (SDM), and MaxEnt calibration and optimization were performed to select the best candidate model. For predicted climate scenarios, the future data is drawn from SSPs 245 and SSPs 585 of the 2050s and 2070s. Out of total 20 variables, annual precipitation, elevation, precipitation of driest month, slope aspect, minimum temperature of coldest month, slope, precipitation of warmest quarter, and temperature annual range (in order) were detected as the most influential drivers. A high accuracy value (AUC-ROC > 0.9) was observed for all the predicted scenarios. The habitat suitability of the targeted species might expand (about 3.7 to 13%) under all the future climate change scenarios. The same is evident according to local residents as species which are locally considered extinct in most of the area, might be shifting northwards along the elevation gradient away from human settlements. This study recommends additional research is conducted to prevent potential population collapses, and to identify other possible causes of local extinction events. Our findings will aid in formulating conservation plans for the Himalayan goral in a changing climate and serve as a basis for future monitoring of the species.

## 1. Introduction

The Himalayan ecosystem is highly susceptible to climate change, especially global warming [1]. Global hotspots of biodiversity, with high levels of species diversity and a large number of endemics, are facing the enormous threat of species extinction [2]. A recent report on global biodiversity states that nearly one million species of plants and animals are facing the threat of extinction [3]. According to the latest IUCN Red List, approximately 42,100 species are classified as threatened, making up nearly one-third of all assessed species [4]. In general, endemic species with limited geographical arrays are at higher risk of extinction than those with wide geographic range [5]. The primary causes of species extinction on a global scale are changes in climate, land use, excessive exploitation, poaching, pollution, and invasive alien species [6]. The ongoing threat to global biodiversity posed by these factors is thought to be climate change [7]. The sustained solution to address both climate change and the biodiversity crisis is believed to be the implementation of nature-based solutions that protect natural ecosystems and recover species [8].

When implementing recovery efforts, it is critical to give priority to disturbed landscapes that could enable biodiversity conservation, especially of endemic species which are more vulnerable to extinction [9]. As a result of climate change, it can be challenging for conservation managers to decide where to restore and how to recover when attempting to restore species. Species distribution modeling (SDM), a tool that determines suitable habitats and predicts range shifts under climate change projections, presents an encouraging approach to plan recovery methods for endangered endemic species in regions of high biodiversity (e.g., the Himalaya) [10]. SDM methods have recently been utilized in fields such as invasion biology, restoration ecology, and conservation ecology to forecast hotspots for the protection and cultivation of endemic and threatened taxa [11,12]. The SDM method, based on the niche conservatism theory, predicts the distribution of species along spatio-temporal gradients using a combination of climatic and other environmental variables with data on species distribution [13]. The influence of future climate change on a region of appropriate habitat for a species is frequently predicted using the niche modeling technique known as the MaxEnt (maximum entropy) model [14]. The MaxEnt model can accurately forecast a small number of species distribution ranges, making it ideal for making predictions about some threatened or endemic animals. The first challenge, to determine where to restore, can be addressed by using the projected appropriate locations as potential locations for the vulnerable species’ recovery [15]. The SDM method for identifying suitable sites considering climate only is inadequate for successful species recovery. To overcome this limitation, it is essential to evaluate the biotic and microhabitat conditions at the SDM-determined habitats as well. This will address the second challenge in recovery ecology [16]. Therefore, combining previously largely unrelated areas of research like macro-spatial SDM insights with local-scale ecology could aid in creating effective recovery strategies.

The Himalayan goral (HG) is endemic to the Himalayan Mountains in Asia, including southern Tibet in China, Bhutan, northern India (Himachal Pradesh, Arunachal Pradesh, Jammu and Kashmir, Sikkim, and Uttarakhand), northern Pakistan, Nepal, and possibly western Myanmar [17,18,19]. The subspecies *N. goral bedfordi* occurs in Himachal Pradesh, Jammu and Kashmir, Uttaranchal, and *N. goral* in Arunachal Pradesh and Sikkim [17]. Himalayan goral (*N. goral bedfordi*) is listed as Near Threatened [20]. Furthermore, fossil records indicate that the current Southeast Asian caprine populations represent relict groups that were once more widespread during the Pleistocene era but became limited to highlands and high-altitude mountain ranges during the Holocene interglacial period. This suggests that these caprines were abundant in tropical lowlands and may have exhibited habitat partitioning in past coexistence areas, given their close genetic relatedness [21]. High potential sites for goral distribution in Jammu and Kashmir along the Pirpanjal and Ka-zi-Nag Range comprise Shamshabari, Kazinag National Park, Limber Wildlife Sanctuary, Lachipora Wildlife Sanctuary, Naganari Conservation Reserve, Ta-takuti-Kalamuund Wildlife Sanctuary, and Khara Gali Conservation Reserve. [17,18].

The term “Himalaya” means “the abode of snow” in Sanskrit. The Himalayas were created around 50 million years ago as a result of the Indian and Eurasian plates colliding, according to the continental drift theory [22]. As a result of the collision between India and Eurasia, a complex geological wedge has been formed, where three tectonic domains have been added to the structure in a north-to-south direction. These domains are known as the High Himalaya, the Lesser Himalaya, and the Sub-Himalaya [23]. The Himalayas is warming at a relatively faster rate (0.06 °C/year) compared to the rest of the globe [24], which raises the possibility of species extinction in the area. The region may become a hotspot for species losses as a result of human and climate-related risks to wildlife. It is predicted that the Himalayas’ rapidly changing climate will increase the extinction risks in the near future, especially for endemic and threatened species. As a result, it is necessary to integrate threats caused by climate change into policies for conserving nature and species recovery practices.

This study used MaxEnt modeling to identify the Himalayan goral’s current appropriate habitats over the entire Himalayan range and estimate how their range might change in relation to various predicted future climate change scenarios. To address the recovery issues, this study asks the following questions: 1. Where are the suitable microhabitats for the Himalayan goral in the Himalayas under current and future climate change scenarios, and what are the leading environmental variables? 2. How will the predicted distribution range of the species respond under different future climate change scenarios? 3. How can the SDM predictions be used to guide the recovery efforts for Himalayan goral in the study area?

## 2. Materials and Methods

### 2.1. Study Area

**Figure 1:**
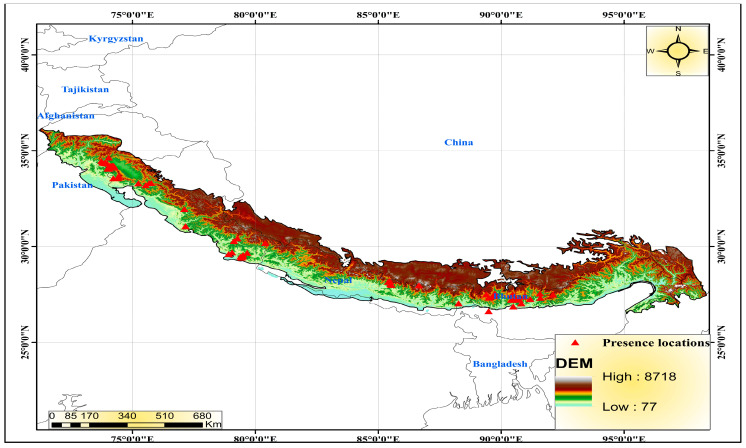
Study area showing the Himalayan mountain range and recorded presence points of the Himalayan goral in South Asia.

The Himalayas, meaning “Abode of Snow,” is a relatively young mountain range separating the Indo-Gangetic Plain and the Tibetan Plateau. This range spans nearly 2400 km in length from Afghanistan in the west to Burma in the east (27–36° N latitude and 72-91° E longitude), it connects the Near East and Central Asia with East Asia (Figure 1). The range is shared by five countries: Nepal, India, China (Tibet), Bhutan, and Pakistan, with most of it being in the first three.

### 2.2. Species Presence Data

To gather the species presence data, Himalayan goral potential occurrence sites in the study area were identified using field surveys, literature, and the GBIF (Global Biodiversity Information Facility) database. From the GBIF and published data, records of species distribution from 1864 to 2019 were chosen, and duplicate records were eliminated. To avoid the spatial pseudo-replication, only GPS (global positioning system) locations with an accuracy of 15 m were selected and filtered to keep one presence point within each of 5 km^2^ pixel based on raster data resolution (i.e., 2.5 arc-min).

We conducted field surveys between the years 2015 and 2021 to record the Himalayan goral presence. The goral was observed with the aid of binoculars (8 × 40) and a spotting scope (15 × 45) from trails and vantage points across the potential habitat. Sightings and signs were recorded with the habitat features distance to the nearest cliff and slope attributes of ruggedness for every sighting. Locations and elevation were recorded using a handheld GPS. A questionnaire survey was prepared to interview knowledgeable local persons and various department staff about their knowledge of the possible presence of goral in the area. The observers’ knowledge was assessed through one to one interaction and by discussing the features of goral. A total of 81 distinct location points of the targeted species were used to build the SDM.

### 2.3. Environmental Data Collection and Variable Selection

A total of 19 bioclimatic variables (resolution: 2.5 arc-min) and elevation data (resolution 30 arc-s) was obtained from the WorlClim (ver. 2.1) (www.worldclim.org, accessed on 15 June 2022). Elevation data was further processed in ArcGIS to generate the slope and aspect variables. Additionally, the topographic data was resampled to bioclimatic data resolution. For future simulations, two shared socio-economic pathways (SSPs) including SSPs 245 and SSPs 585 of two time spans including 2041–2060 = 2050s, and 2061–2080 = 2070s of Coupled Model Intercomparison Project, Phase 6 (CMIP6) and belonging to Global Climate Model of BCC-CSM2-MR (resolution: 2.5 arc-min) were downloaded and used.

The distribution of Himalayan goral is influenced by various factors including temperature, precipitation, geographical barriers, geological formations, and other biological variables [25]. Our model used a total of 19 bioclimatic and three biophysical variables (elevation, slope, and aspect) to assess the environmental factors impacting species distribution.

To ensure independence and eliminate spatially correlated data points, this study employed the following two-step procedure. First, a preliminary model (with default setting) was run to assess the variables contribution. A threshold value of >1% was used to screen the variables. In the second step, the remaining variables (above contribution threshold) were analyzed for a pairwise Pearson’s correlation (r). A threshold value (r ≥ ±0.8) was used to further reduce the number of variables. Any two variables with an r value above the selected threshold were targeted, and those contributing less were omitted [26,27,28].

### 2.4. Preliminary Varaibles Processing

**Figure 2:**
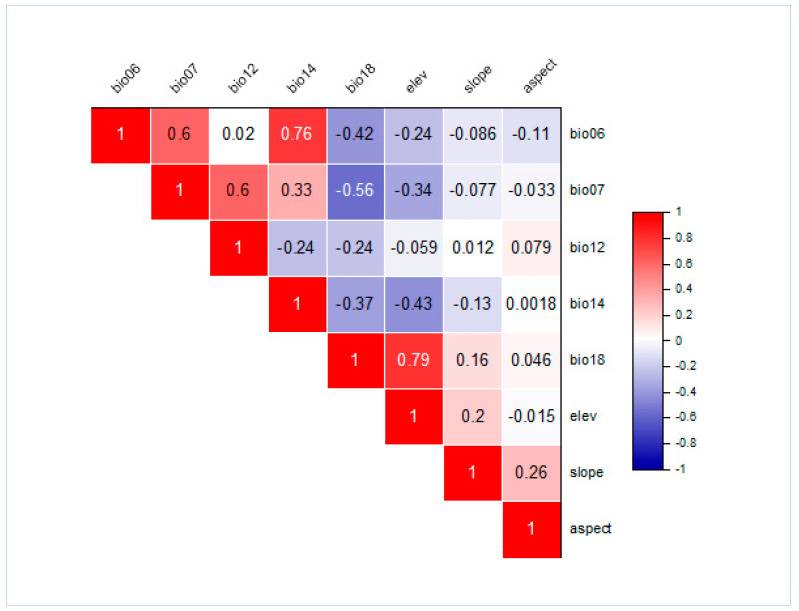
Pearson correlation heat map showing the pairwise correlation of climatic and biophysical variables (threshold; r = ±0.8) used in the distribution modeling of Himalayan goral.

The threshold application and Pearson’s correlation coefficient resulted in a total of eight crucial bioclimatic and topographic variables. These include minimum temperature of coldest month, annual precipitation, precipitation of warmest quarter, annual temperature range, precipitation of driest month, aspect, slope, and elevation [29]. The pairwise correlation of the finally selected variables is also presented (Figure 2).

### 2.5. Model Calibration and Optimization

MaxEnt prediction model calibration and optimization is crucial in SDM to select the best candidate model. Selecting different regularization multiplier (RM) values and feature classes (FC) are generally required to tune the model for better prediction reliability. The optimal MaxEnt model settings were identified based on threshold-dependent (i.e., omission rate) evaluation metrics, in order to prevent overfitting and improve model transferability. For this, multiple combinations of both RM values (a total of eight different values with range: 1–4, and interval: 0.5) and six FCs (L, LQ, H, LQH, LQHP, and LQHPT where L = Linear, Q = Quadratic, H = Hinge, P = Product, T = Threshold) were targeted [30]. The ENMEval package in R was used to build the bias file using occurrence and environmental data to be used in the model.

The data for this study was analyzed using MaxEnt 3.4.4. to forecast the optimal habitats for Himalayan goral in the study region [31]. The MaxEnt model, based on ecological niche theory, predicts the possible distribution of a target species within a research area using presence data information [32]. MaxEnt software uses species presence data to identify the most suitable habitats for the target species with high accuracy [31] and it is one of the most advanced and promising SDM techniques [33,34]. MaxEnt has overtaken other approaches used for the aforementioned reasons due to its superior prediction accuracy [35,36].

We applied MaxEnt, a highly accurate machine-learning species distribution modeling approach, to model the connection between wildlife distributions and environmental conditions [36], to create models of the interactions between the species occurrences and climatic factors. The other MaxEnt settings for better prediction accuracy and model performance include use of 10th percentile presence probability of the species, 10-fold cross-validation method, complementary log-log (clog-log) output format, 10,000 background points, 10 replicate runs, 500 iterations, response curves development, and testing of jackknife importance in all the final optimized SDMs.

### 2.6. Model Evaluation and Preditions Reclassification

The evaluation of the optimized SDMs was assessed using area under curve (AUC) of the receiver-operator characteristic (ROC) curve values. The AUC-ROC value of >0.9 was considered an excellent model for predictive performance [31,33,37,38]. The AUC score evaluates the model’s capacity to discriminate between changes in species distribution under potential future climatic scenarios and reflects how well the model fits the test data [31,37]. AUC values closer to 1.0 indicate that the model performs better than chance, whilst 0.5 indicates that the model performs no better than chance [37]. The averaged MaxEnt prediction output for the possible existence of Himalayan goral in the study area varied from 0–1. The modeled outputs were classified into a total of four habitat suitability levels: 1. 0–0.2 not suitable habitat; 2. 0.21–0.5 low suitability habitat; 3. 0.51–0.8 moderately suitable habitat, and 4. 0.81–1 highly suitable habitat [39,40,41,42].

## 3. Results

### 3.1. Model Evaluation

**Figure 3:**
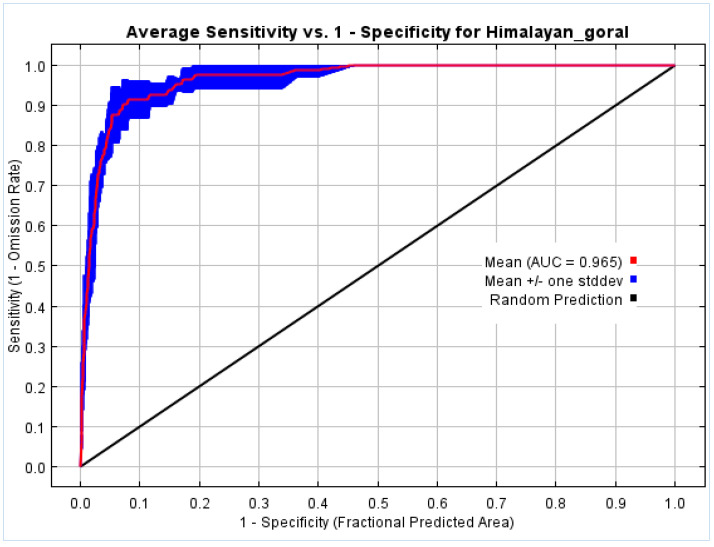
Graph depicting AUC-ROC value of the current climate (1970s–2000s) MaxEnt model. The model’s precision was 0.965.

MaxEnt generated outputs for 10 replications, which were then averaged into a single model prediction, along with response curves and the AUC graph. The average model was used to evaluate prospective migration paths and make suitability assumptions. Our findings suggest that the use of LQH as FCs in combination with an RM value of 1.5 produced the optimal candidate model with an AUC score of 0.965. The projected range of the Himalayan goral in the whole Himalaya region received a predictive habitat suitability score ranging from 0 to 0.96. The AUC value of the MaxEnt model can be used directly as a standard for model prediction performance because it can calculate the ROC curve and draw it on its own. The expected outcomes of this study’s ROC curve are displayed in Figure 3. According to the findings of the curve analysis, the model’s average AUC value was 0.965, which was much higher than the AUC value of the random prediction model (0.5), indicating that the predicted outcomes had a better degree of accuracy. This meant that the model’s anticipated distribution area and the species’ actual distribution area were a good fit.

### 3.2. Environmental Variables Responsible for the Distribution

**Figure 4:**
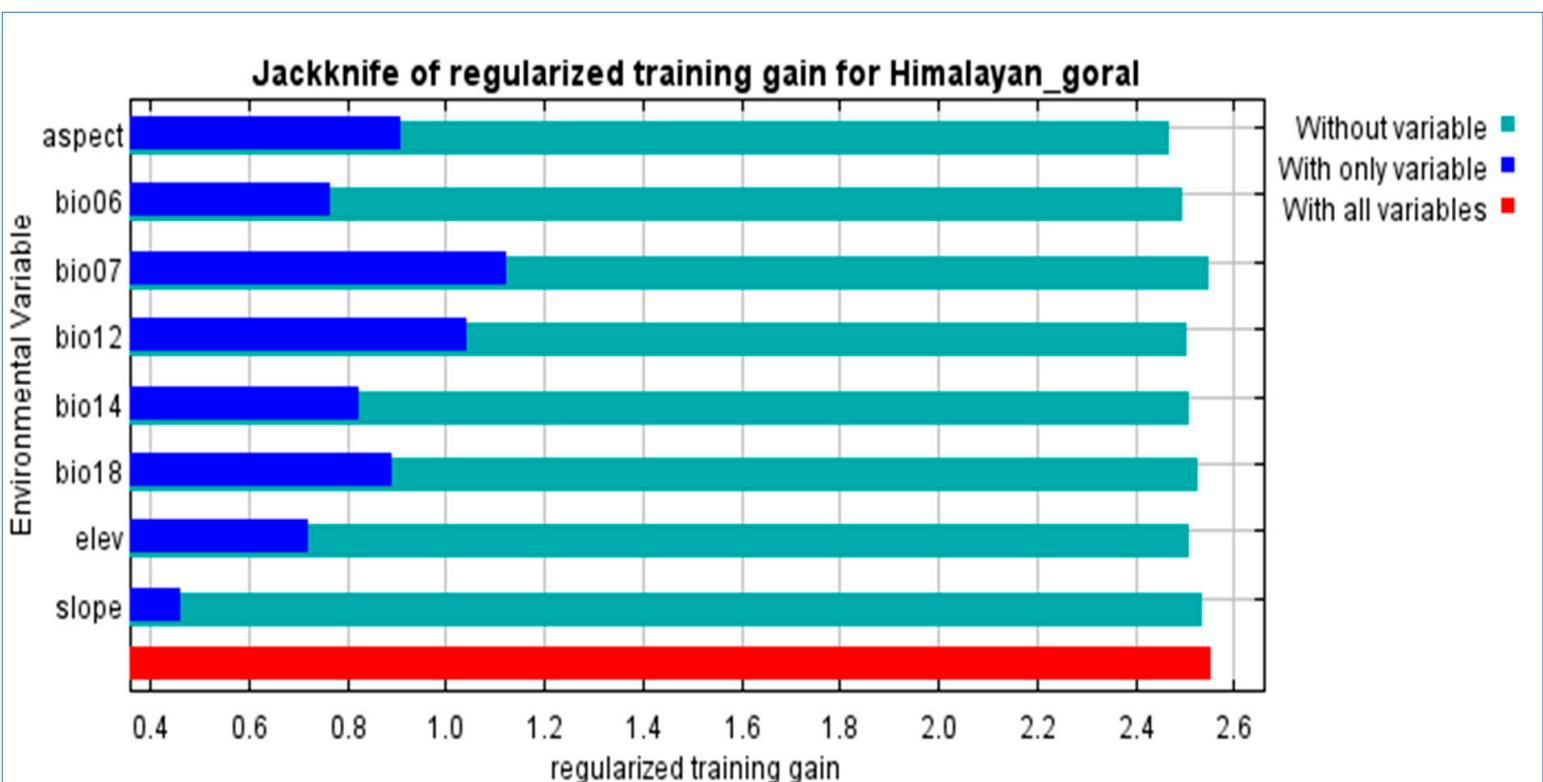
MaxEnt model for Himalayan goral using the jackknife of regularized training gain to determine the predictive strength of environmental variables.

**Figure 5:**
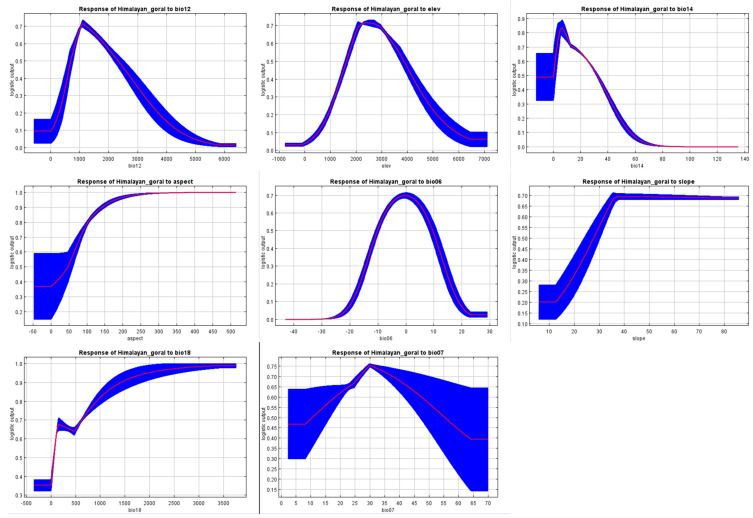
Response curves of highly contributing variable in the MaxEnt distribution modeling of Himalayan goral. Bio 12 (annual precipitation); elevation; bio 14 (precipitation of driest month); aspect; bio 06 (minimum temperature of the coldest month); slope; bio 18 (precipitation of warmest quarter); bio 07 (temperature annual range).

The variables with a higher contribution percentage for habitat suitability of Himalayan goral includes annual precipitation, elevation, precipitation of driest month, aspect, and minimum temperature of coldest month. The variables with relatively low contribution in the SDMs of Himalayan goral included slope, precipitation of warmest quarter, and temperature annual range (Table 1). The findings revealed that the way the chosen variables characterize Himalayan goral current distribution was excellent. The jackknife test showed that the MaxEnt model’s contributions to the distribution of Himalayan goral were 38.2% and 32.2% for the annual precipitation (Bio12) and the elevation, respectively (Figure 4). We undertook a single study to show the influence of Bio12 and elevation as a single factor because of their highest contribution rates. The chance of Himalayan goral presence increased initially and then declined as annual precipitation increased (Figure 5). The probability was greater than 0.6 when annual precipitation was between the ranges of 700–1000 mm, indicating a very appropriate location, whereas the chance of presence peaked at around 1200 mm, or about 0.7. The elevation for highly suitable habitats ranged from 2000–3000 m (Figure 5). 

### 3.3. Current Distribution

**Figure 6:**
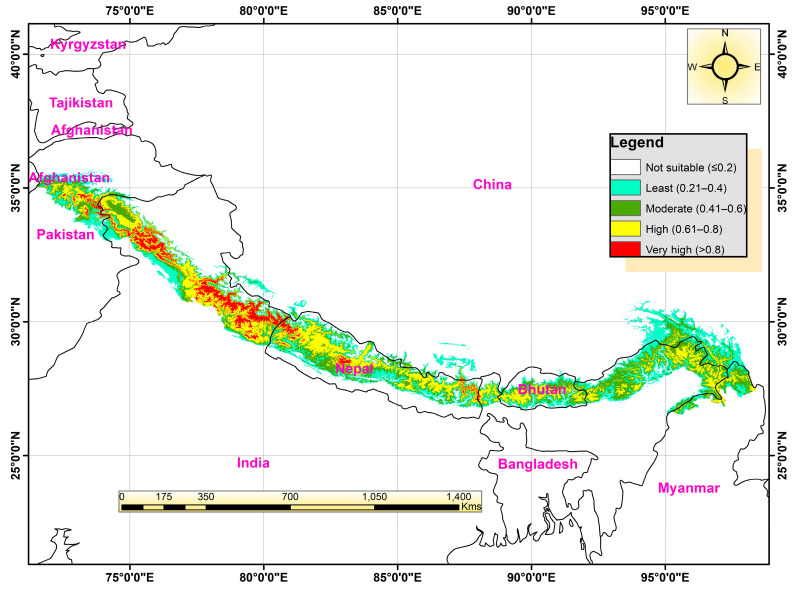
MaxEnt prediction map displaying the potential habitat suitability classification of Himalayan goral in the study area under current climatic (1970s–2000s) conditions.

The Himalayan range encompasses seven Asian countries, including India. It occupies an area of (about) 2500 km^2^ over 11 states and Jammu and Kashmir (J&K) in India (Figure 1). The current suitable locations of Himalayan goral were obtained through a species distribution model that predicted their occurrence (Figure 6). The Himalayan goral is thought to reside in the Himalayan region that stretches from Nepal to northwest India, crossing via the J&K region to reach Afghanistan. However, the necessary habitats are not evenly distributed throughout the upper Himalayas. We found that high suitable and medium suitable habitats are spread in patches throughout the Himalayas from north-eastern Afghanistan to central Nepal. Along the Pakistan-Afghanistan border, in the Uttarakhand, Kashmir region, and in the Annapurna region are continuous stripes of highly suitable habitats. 

### 3.4. Potential Habitat Suitability under Future Climate Change Scenarios

**Figure 7:**
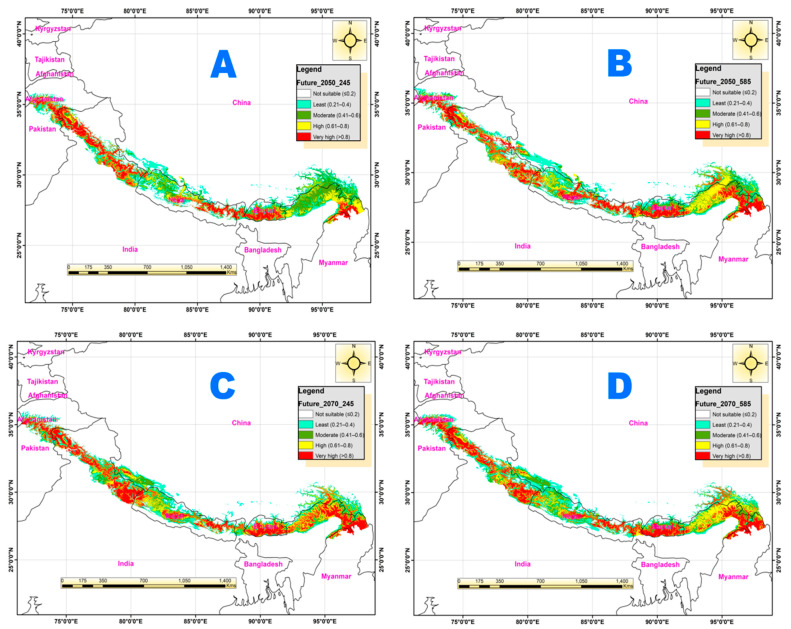
MaxEnt prediction maps displaying the habitat suitability classes under future climate change scenario (**A** = SSPs-245 of 2050s; **B** = SSPs-585 of 2050s; **C** = SSPs-245 of 2070s; **D** = SSPs-585 of 2070s).

When future climate change possibilities were considered collectively, we found that existing most suitable habitat is disappearing and shifting northward as well as along the elevation gradient. In future, in all four SSPs scenarios of the 2050s and 2070s, the predicted suitable habitat might shift northward in Jammu and Kashmir, Pakistan, Uttarakhand, and Nepal, resulting in an uninterrupted distribution of extremely appropriate locations in the north section of J&K territory and their adjacent areas (Figure 7). In four scenarios, however, the availability of suitable habitat is limited. The majority of the appropriate location would be accessible in west Himachal Pradesh and Uttarakhand and moreover their surrounding region in Tibet beside the boundary of the three nations by the year 2050 (SSPs 245 and SSPs 585). The majority of the habitat would vanish between the Nepal area and the Pak-Afghan border in 2050, according to the SSPs 346 scenario, although there would be small strips of appropriate habitat in Himachal between the Kashmir region and Uttarakhand. By 2050, the Kashmir region, the Pak-Afghan border, and the Annapurna region might be devoid of suitable habitat for the Himalayan goral according to the SSPs 245 and SSPs 585 scenarios.

All the predicted highly suitable habitats near the Pakistan-Afghanistan border, Kashmir, and Himachal might be lost by 2070s under all the four considered future climate change scenarios. The Himalayan regions in the west of Uttarakhand, including the Indian, Pakistani, and Afghan Himalayas, might face the disappearance of all the existing or current suitable and highly suitable habitats in the 2050s and 2070s under scenarios SSPs 245 and SPPs 585. It is anticipated that Pakistan, Kashmir, and central Nepal might have only marginally suitable locations in the Pak-Afghan border region between 2050 and 2070.

By 2050, the habitat for Himalayan goral under the SSPs 585 scenario would have expanded more than SSPs 245 and this habitat is anticipated to be dispersed densely over west Nepal, Uttarakhand, and their connected territories in Tibet (Figure 7). By 2070, the habitat would have a similar pattern in the SSPs 245 and SSPs 585 scenarios. However, in SSPs 585 scenarios, the habitat of Himalayan goral would be enlarged in Uttarakhand, west Nepal, and their surrounding territories in Tibet.

## 4. Discussion

Multiple improvements in GIS technology especially during the last two decades have enabled the gathering of diverse spatial and temporal datasets, leading to better understanding of species distribution and the impact of environmental factors at multiple scales [43]. The current study is the first comprehensive analysis of the distribution, habitat appropriateness, and hotspot predictions of the Himalayan goral. According to habitat suitability classes selected in this study (Table 2), least suitable (*p* 0.21–0.4), moderately suitable (*p* 0.41–0.6), and highly suitable (*p* 0.61–0.8) areas for the Himalayan goral are predicted to shrink, whereas very high suitability areas (*p* 0.81–1.0) are predicted to expand remarkably in future. This possible shrinkage in some parts might be due to the increasing rate of existing habitat degradation based on accelerated construction, urbanization, and deforestation in the study area. Our model demonstrated exceptional performance, with strong predictive ability and highly significant predictions, as evidenced by an AUC value greater than 0.9 [44,45,46]. Our model’s AUC value for the current climate modeling was 0.965, indicating that it accurately and thoroughly characterized the Himalayan goral habitat, and significantly distinguished between the possible presence and absence of the targeted species in the Himalayan geographic range. The most recent assessment of habitat appropriateness was perfectly in line with the analysis’ utilization of the available occurrence records. Because annual precipitation (BIO 12) and minimum temperature of the coldest month (BIO 6) were two of the top bioclimate variables contributors to the model, precipitation was the most influential variable on the distribution of Himalayan goral. This is consistent with [47], who stated that annual precipitation (BIO 12) was the main factor influencing the spread of Himalayan goral found in Pakistan. Similarly, elevation was the other key characteristic to predict its habitat. The MaxEnt model results predicted that the most optimal elevation range for the Himalayan goral varies from 2000 to 3000 m above sea level (ASL). This is consistent with what is known about the species, as it is typically found at extreme elevations ranging from 1000 to 4000 m ASL. Within this elevation range, the goral may prefer areas with a mix of vegetation types, including open grasslands, shrublands, and forests. The availability of water sources, such as streams or springs, may also be an important factor in habitat selection, and directly depend on precipitation type, pattern, and amount in the study area. Many researchers reported a similar altitude range as the ideal habitat for goral in India and other native neighboring regions [43,48,49,50].

Therefore, elevation is the key factor influencing the distribution of the Himalayan goral. The SDM results in this study are aligned accordingly, and elevation is detected as the second-highest contributor, which agrees with [43], who identified altitude as one of the key elements of Himalayan goral habitat that sets it apart from other ungulates. However, the elevation range between 2000 and 3000 m ASL is regarded as the most favorable as an appropriate microhabitat in the study area. However, the local residents suggested that the Himalayan goral is probably shifting further upward along the elevation and trying to avoid the growing heat and anthropogenic disturbances. Our research confirms [43] findings that the preferred habitat for the Himalayan goral in Pakistan is the upper mid-hill to high-mountain region (more than 2000 m ASL). Under both SSPs 245 and 585 scenarios, it is projected that the Himalayan goral habitat will experience an overlap in the future, with a significant proportion of the habitat shifting. The model also showed that any future shift in habitat along the elevation gradient might mostly lean longitudinally rather than latitudinally in the study area. This demonstrates that the Himalayan goral is sedentary by nature and stays within a predetermined home range throughout the year.

In order to create efficient conservation strategies, it is essential to comprehend how animals react to climate change [51,52]. The direct effects of climate change on the ecosystems of diverse species have already been highlighted in numerous studies [51,53,54]. The current study presents the first extensive evaluation of how the distribution of Himalayan goral may vary under climate change scenarios. We used the MaxEnt modeling approach to cover the entire Himalayan region where the species is known to exist, and predicted both the current and future possible distribution using the MaxEnt modeling technique and a range of greenhouse gas emission scenarios over a number of time periods. Under SSPs 245, the currently suitable habitats decreased and shifted to eastern Himalayan regions in the years 2050 and 2070, while both the range and the suitable habitat decreased under SSPs 245 and SSPs 585 scenarios (2050s and 2070s). The distribution of large mammals is affected by climate change, but not uniformly across species. The expected habitat fluctuation during climate change differs depending on the species. The findings of [55] reveal that African mammals respond differently to scenarios of global climate change, with some species losing nearly all of their viable habitats while others remained steady or even gained new habitats. Our findings are consistent with these studies [56,57].

Our findings demonstrated that the regional distribution of Himalayan goral would decrease under the predicted climatic circumstances for the years 2050 and 2070 under the two tested SSPs (245 and 585). The simulations showed that when this species disappears from human view, the habitat range and core habitats might shift in future. The environmental envelope (precipitation and temperature) of the Himalayan goral will become less conducive to its existence, which might be the cause of the range shift. The Himalayan goral lives on isolated patches in the study area, making them more vulnerable to climate change since species with small ecological niches are more susceptible to climate change than species with broader ecological niches [58,59].

Despite their undeniable significance in defining the geographic range of many species, it is interesting to note that climatic influences are not the only ones causing species to disperse throughout any land area [14]. To forecast a more accurate image of the spatial distribution of the species, other ecological aspects such as ecological interactions, dispersal pattern and capability, habitat preference, resource distribution, and availability merit careful investigation and improved integration within SDMs [36]. The inability to completely incorporate the species ecology ideas into the modeling process due to incomplete data has added some constraints to this work. Sample size, sampling bias, the geographical resolution of the predictors, including their selections, and multi-collinearity issues are additional elements that affect the SDM’s limitations and uncertainties. These issues should be carefully considered during the modeling process [60,61]. However, MaxEnt is considered as the most appropriate tool due to its outstanding predictive strength and ability to function admirably with tiny sample sizes. Although this study tried to address the multi-collinearity problems (by removing the variables that are strongly correlated) and background sampling bias (by just using background samples from the region of occurrence records), we recognize that there may still be some uncertainty in the results as biotic interactions and dispersal capacity were not targeted. The estimated potential distribution maps of the species’ suitable habitats should be further refined in future by including the physiologically pertinent parameters in the modeling process. The predictions presented in this study for the region might warrant some serious consideration for species conservation in light of climate change, especially global warming.

The current study is the first to describe the link between ecological parameters and habitat viability, anticipate changes in habitat suitability due to predicted climate change, and model the Himalayan goral in its distributional range. The findings are in line with earlier research on climate change in the same ecosystems, and demonstrate that the habitat appropriateness of the Himalayan goral is substantially related to climate in the area [62,63,64]. With a lack of research on Himalayan goral distribution in the study area, the findings of this study strongly support additional investigation. For conservation strategies and plans, it is strongly advised to announce the expected suitable areas as nature reserves to house more diversity with few risks in both the existing and anticipated future climatic conditions.

The extinction of local species is considered a leading cause of alterations in the structure and function of the ecosystem in any area [65,66,67,68]. Our research area includes multiple biodiversity hotspots, contains a high percentage of vulnerable species, and has seen an increase in recent years in the risk of extinction, mostly as a result of habitat degradation and illegal hunting [69,70,71,72]. We have assessed and mapped the currently acceptable habitat for this species as well as projections for it under scenarios of a warming climate and changing land use. Our study also offers opportunities for protecting numerous other species that share its habitat. To help reduce biodiversity loss due to habitat degradation and loss, for instance, our suitable location map and forecasting could be utilized to pinpoint crucial habitat areas requiring instant conservation efforts. Additionally, our high-resolution maps could assist park managers and conservationists in identifying and prioritizing conservation efforts for crucial habitats, focusing on preserving threatened remnant patches and critical connecting habitats that are forecasted to vanish in the future.

Understanding the influence of climate change and human threats on Himalayan goral distribution is crucial for managing such species and mitigating potential threats. Our research provides a preliminary evaluation of the effect of climate change on the distribution of HG in its primary habitats in the Himalayan region. This assessment could aid in future analyses of HG distribution across its entire range in the Himalayas and be helpful in creating any management strategies for wildlife conservation in the face of climate change.

## 5. Conclusions

The current study is the first to identify Himalayan goral (HG) habitat suitability variations under multiple future climate change scenarios in its native range using the MaxEnt modeling tool. The two main environmental factors, Bio12 and elevation, were found to have major effects on HG distribution. Overall, the suitable habitat might shrink under the predicted 2050s and 2070s future climate change scenarios. The current modeling framework demonstrated habitat suitability under both the present climate and future climate change predictions based on various possible greenhouse gas emission scenarios. This study might serve as a baseline work and be useful in developing and putting into practice conservation strategies for the HG in order to lessen the effects of climate change and human disturbance. This study suggests that such an approach might make it possible to undertake more habitat assessments in conditions where semi-structured field surveys are feasible but adequate funding and statistical know-how are not immediately available.

## Figures and Tables

**Table 1:** Environmental factors selected after preliminary processing and their contribution rates.

Description	Code	Percent Contribution
Annual Precipitation	bio12	38.2
Elevation	elev	32.2
Precipitation of Driest Month	bio14	11
Aspect	spect	8.2
Min. Temperature of Coldest Month	bio06	4.6
Slope	slope	2.5
Precipitation of Warmest Quarter	bio18	2.1
Temperature Annual Range	bio07	1.3

**Table 2:** The predicted probability of habitat suitability of the Himalayan goral under different climate change scenarios.

Climate Change Scenario	Predicted Probability of Occurrence of Himalayan Goral under Considered Habitat Suitability Classes					
	Not Suitable	Least	Moderate	High	Very High	Total Suitable Land Area (km^2^)
	(*p* ≤ 0.2)	(*p* 0.21–0.4)	(*p* 0.41–0.6)	(*p* 0.61–0.8)	(*p* ≥ 0.81)	
**Current climate**	515,799	25,200	24,113	23,595	6294	79,201
**SSPs_245_2050**	512,854	21,129	24,781	15,775	20,461	82,146
**Rate of change (%)**	−0.6	−17.6	2.7	−40.3	117.9	3.7
**SSPs_585_2050**	505,989	21,810	19,076	20,171	27,953	89,011
**Rate of change (%)**	−1.9	−14.4	−23.4	−15.7	149.1	11.7
**SSPs_245_2070**	508,792	19,319	17,182	19,642	30,065	86,208
**Rate of change (%)**	−1.4	−26.6	−33.9	−18.3	156.4	8.5
**SSPs_585_2070**	504,857	21,034	19,829	21,006	28,275	90,143
**Rate of change (%)**	−2.14	−18.07	−19.56	−11.62	150.24	12.94

## Data Availability

All data are available within this publication.
